# Unilateral Pleural Effusion as an Initial Manifestation of Systemic Lupus Erythematosus in a Patient of Advanced Age

**DOI:** 10.7759/cureus.63327

**Published:** 2024-06-27

**Authors:** Naoaki Tsuji, Noeru Inoguchi, Takenori Sakai, Taisei Furumuro, Kiho Takaya

**Affiliations:** 1 Pulmonology, Otsu City Hospital, Otsu, JPN; 2 Internal Medicine, Otsu City Hospital, Otsu, JPN

**Keywords:** diagnosis, autoimmune disease, late-onset systemic lupus erythematosus, pleural effusion, advanced age, systemic lupus erythematosus disease

## Abstract

Systemic lupus erythematosus (SLE) is a chronic, multisystem autoimmune disease that can manifest in older individuals, presenting unique diagnostic challenges because of its atypical presentations and comorbidities. Pleural effusion is a relatively uncommon manifestation of SLE, with studies suggesting a higher prevalence in older than younger patients. We herein report an atypical case of delayed-onset SLE in a 75-year-old man with left-sided pleural effusion as the initial presentation. This case underscores the difficulty of diagnosing SLE in patients of advanced age and the importance of considering a broad range of differential diagnoses, even in cases that may suggest a more common disease. This case also highlights the fact that unilateral pleural effusion can be an initial manifestation of SLE, and when the cause of the pleural effusion is unclear, SLE should be considered as a potential diagnosis.

## Introduction

Systemic lupus erythematosus (SLE) is a chronic, multisystem autoimmune disease characterized by the production of autoantibodies targeting the body’s own tissues. Although it typically affects younger women, SLE can also manifest in older individuals. In such cases, it presents unique diagnostic challenges because of its atypical presentation and the presence of comorbidities that can obscure the underlying autoimmune process [1]. Pleural effusion, defined as fluid accumulation in the pleural space, is a relatively uncommon manifestation of SLE. Interestingly, studies suggest that the incidence of pleural effusion increases with age, with a higher prevalence observed in older than younger patients with SLE. This observation highlights the potential for pleural effusion to serve as a presenting symptom in older individuals with SLE. We herein report a remarkable case of delayed-onset SLE with unilateral pleural effusion as the initial presentation. This is, to the best of our knowledge, an extremely rare case of SLE in a patient of advanced age presenting exclusively with unilateral pleural effusion, representing a significant contribution to the medical literature. This case underscores the difficulty of diagnosing SLE in older patients and the importance of considering a broad range of differential diagnoses, even in cases that may suggest a more common disease.

## Case presentation

A 75-year-old man with no significant medical history or current medications was referred to our department of respiratory medicine by his primary care physician for further evaluation of left-sided pleural effusion. He reported a one-month history of pleuritic chest pain on the left side when in the supine position. The patient denied a history of smoking and had no family history of autoimmune disease. On physical examination, the patient presented with a blood pressure of 124/81 mmHg, a regular pulse rate of 101 beats per minute, a body temperature of 36.9°C, and a peripheral arterial oxygen saturation of 98% on room air. He showed no signs of peripheral lymphadenopathy, skin rash, edema, or arthralgia.

**Figure 1:**
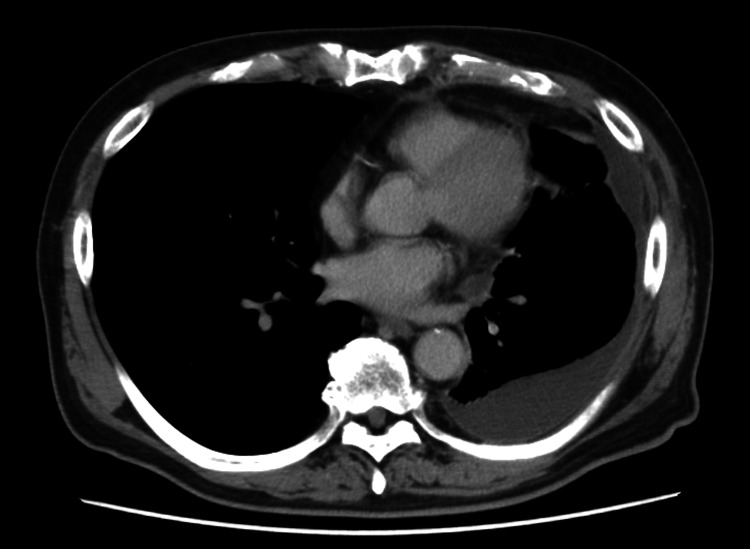
Contrast-enhanced chest computed tomography performed at presentation revealed left pleural effusion.

Laboratory tests revealed mild normocytic anemia (hemoglobin, 11.7 g/dL; mean corpuscular volume, 94.3 fL) and an elevated C-reactive protein (CRP) level of 6.33 mg/dL (Table 1). The platelet count, lymphocyte count, and renal function tests were within normal limits. A T-SPOT test was performed to check for tuberculosis, but the results were negative. Tumor markers, including carcinoembryonic antigen and pro-gastrin-releasing peptide, were also negative. A chest computed tomography scan showed a small amount of pleural effusion at the left lung base without evidence of cardiac enlargement (Figure 1). Echocardiography revealed no abnormalities. Two sets of blood cultures were negative. The pleural fluid was exudative, had a pH of 7.22, and contained 40% neutrophils and 37% lymphocytes (Table 2). The pleural fluid adenosine deaminase level was low, and a pleural fluid culture was negative. Pleural fluid cytology was also negative for malignant cells. At the initial presentation, there was no suspicion of SLE; therefore, antinuclear antibodies and complement levels were not assessed. Pleural fluid analysis revealed mild neutrophilic predominance, raising our suspicion for both empyema and parapneumonic effusion, common causes of pleural effusion in this patient population. Concurrent evaluation for malignancy and tuberculosis yielded negative results. The patient was treated with lascufloxacin, a fluoroquinolone antibiotic, for one week in-hospital and for an additional three weeks as an outpatient. During follow-up, his inflammatory marker concentrations, including a gradual decline in CRP levels to 1.75 mg/dL, and pleural effusion volume decreased, and his symptoms improved. This improvement led to the misdiagnosis of infectious diseases such as empyema during the healing phase. The antibiotics were then discontinued.

**Table 1:** Blood and urine test findings.

Test	Result	Normal range
White blood count (WBC)	6.7x10^3^/uL	4.00 to 11.00 x 10^3^/uL
Absolute lymphocyte count	0.9x10^3^/uL	1.00 to 4.00 x 10^3^/uL
Hemoglobin (Hb)	11.7 g/dl	12 to 16 g/dl
Platelet	331x10^3^/uL	150 to 450x10^3^/uL
Serum creatinine	0.82 mg/dl	0.5 to 1.1 mg/dl
Serum sodium	137 mmol/L	136 to 145 mmol/L
Serum potassium	3.9 mmol/L	3.5 to 5.0 mmol/L
Total protein	8.1 gm/dL	6.4 to 8.3 gm/dL
Lactate dehydrogenase (LDH)	119 IU/L	135 to 250 IU/L
C-reactive protein (CRP)	6.33 mg/dl	0 to 5 mg/dl
Carcinoembryonic antigen (CEA)	1.9 ng/ml	0 to 5 mg/ml
Pro-gastrin-releasing peptide (proGRP)	41.1 pg/ml	0 to 80.9 pg/ml
B-type natriuretic peptide (BNP)	11.9 pg/ml	0 to 18.4 pg/ml
T-SPOT.TB Test	Negative	Negative
Urine, protein	Negative	Negative
Urine red blood cells (RBCs)	0 /hpf	0 to 5 /hpf

**Table 2:** Pleural effusion findings.

Test	Result	Normal range
pH	7.22	7.38-7.42
Lactate (Lac)	5.4 mmol/L	<2.0 mmol/L
Glucose (Glu)	57 mg/dL	70-100 mg/dL
Carcinoembryonic antigen (CEA)	1.3 ng/mL	<3.0 ng/mL
Specific gravity	1.041	<1.015
Cell count	3010 cells/μL	<1000 cells/μL
Neutrophils	40%	<25%
Lymphocytes	37%	>50%
Macrophages	2%	<70%
Eosinophils	21%	<10%
Protein	6.1 g/dL	<3.0 g/dL
Glucose	61 mg/dL	70-100 mg/dL
Lactate dehydrogenase (LDH)	723 U/L	<200 U/L
Adenosine deaminase (ADA)	87.1 U/L	<40 U/L

One week following an outpatient follow-up, the patient developed progressive gait ataxia and hand dexterity impairments. He also reported persistent fatigue, worsening lightheadedness, and experienced mild joint pain, particularly in the knees and hands. Despite conservative management by a local physician, the patient’s symptoms exhibited continued deterioration. In addition, the previously resolved pleuritic chest pain recurred. Two months later, the patient presented with poor food intake and newly developed anemia, necessitating readmission to our hospital.

**Figure 2:**
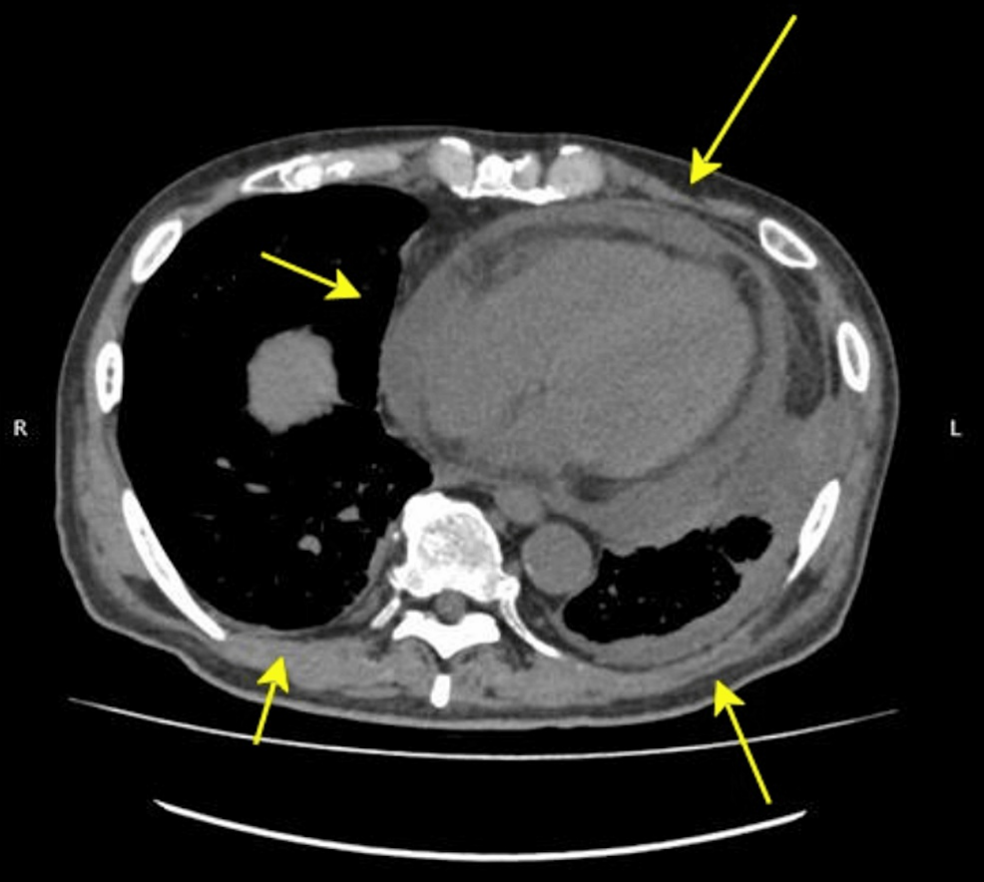
Chest computed tomography revealed bilateral pleural effusion and pericardial effusion.

On admission, the patient exhibited a low-grade fever of 37.8°C, of which he was unaware. Laboratory investigations revealed a hemoglobin concentration of 8.7 g/dL, a leukocyte count of 7,300 cells/µL with lymphopenia of 1,318 cells/µL, an activated partial thromboplastin time of 44 seconds, an elevated CRP concentration of 16.57 mg/dL, and a markedly positive antinuclear antibody titer of 1:10,240 with a homogeneous pattern. In addition, the patient tested positive for RF and SSA antibodies, while the anti-DNA antibody was negative (Table 3). Liver and renal function test results, along with urinalysis results, were unremarkable. Chest computed tomography demonstrated bilateral pleural effusion and pericardial effusion (Figure 2). Alternative diagnoses were deemed improbable considering the clinical presentation, laboratory findings, and imaging results. The patient fulfilled the 2019 European League Against Rheumatism/American College of Rheumatology classification criteria for SLE by meeting the entry criterion of positive antinuclear antibody and the additional criteria of bilateral pleuritis and the presence of arthralgia. Given the low probability of alternative diagnoses and the patient meeting the classification criteria for SLE, we conclude that the patient has SLE. The patient was then transferred to a specialist for further management, and treatment with hydroxychloroquine and steroids was initiated. The patient's symptoms showed a trend toward resolution subsequent to treatment initiation.

**Table 3:** Blood, urine, and culture test results.

Test	Result	Normal range
White blood cell count (WBC)	7.3 x 10³/µL	4.0-11.0 x 10³/µL
Absolute lymphocyte count	0.9 x 10³/µL	1.0-3.0 x 10³/µL
Hemoglobin (Hb)	8.7 g/dL	12-16 g/dL
Platelet count	365 x 10³/µL	150-450 x 10³/µL
Serum creatinine	0.65 mg/dL	0.5-1.1 mg/dL
Serum sodium	130 mmol/L	135-145 mmol/L
Serum potassium	3.6 mmol/L	3.5-5.0 mmol/L
Total protein	7.6 g/dL	6.4-8.3 g/dL
Lactate dehydrogenase (LDH)	180 IU/L	135-250 IU/L
C-reactive protein (CRP)	16.57 mg/dL	0-5 mg/dL
Prothrombin time (PT%)	63%	70-140%
PT-INR	1.19	0.8-1.2
Activated partial thromboplastin time (APTT)	44 seconds	20-40 seconds
C3 complement level	93 mg/dL	73-138 mg/dL
C4 complement level	24 mg/dL	11-31 mg/dL
CH-50 (serum complement activity)	36 U/mL	30-45 U/mL
Anti-nuclear antibody (ANA) titer	10240 (homogeneous)	<1:80 (negative)
Anti-DNA double stranded antibody	4.5 IU/mL	<5.0 IU/mL
Anti-Sm antibody (FEIA)	0.8 U/mL	<5.0 U/mL
Anti-U1-RNP antibody (FEIA)	1.7 U/mL	<5.0 U/mL
Anti-SS-A/Ro antibody (FEIA)	240 U/mL	<7.0 U/mL
Anti-SS-B/La antibody (FEIA)	0.9 U/mL	<7.0 U/mL
Anti-Scl-70 antibody (FEIA)	0.6 U/mL	<7.0 U/mL
Cardiolipin antibody IgG	10.5 GPL-U/mL	<15 GPL-U/mL
Cardiolipin antibody IgM	1.3 MPL-U/mL	<12 MPL-U/mL
Rheumatoid factor	105.1 IU/mL	0-20 IU/mL
Angiotensin-converting enzyme (ACE)	14.1 U/L	7-25 U/L
Urine protein	(-)	Negative
Urine red blood cells (RBC)	1 /hpf	0-5 /hpf
Urine white blood cells (WBC)	1-4 /hpf	0-5 /hpf
Blood and urine cultures	No growth	-

## Discussion

This case highlights several lessons regarding the diagnosis of SLE. First, late-onset SLE can present with various symptoms, including pleural effusion and neurologic manifestations. Second, unilateral pleural effusion can be an initial manifestation of SLE. Finally, SLE should be considered a differential diagnosis when the cause of pleural effusion is unclear. Previous reports suggest that serositis (e.g., pleuritis, pericarditis) is more frequently seen as an initial or early manifestation of late-onset SLE [1]. One study showed that pleuritis was the initial symptom in 27% of patients diagnosed with late-onset SLE at the age of ≥50 years [2]. In our patient, unilateral pleural effusion was the chief complaint. SLE-associated pleural effusion is typically exudative [3,4] and can be associated with an elevated CRP level [3], as observed in this case. It has been suggested that small amounts of pleural effusion may resolve spontaneously [5]. Consistent with this, the pleural effusion in our patient resolved naturally.

Empyema is primarily associated with preceding bacterial pneumonia, which is the most significant risk factor for the development of empyema [6]. Other risk factors include immunosuppressive conditions such as diabetes and alcohol dependency [6]. In the present case, the absence of any such risk factors was notable. Despite this, the reduction in inflammatory markers following antibiotic treatment led to a misdiagnosis, assuming that the patient was in the healing phase of empyema. In some cases, young women with unilateral pleural effusion, negative pleural fluid culture, and a clinical picture suggestive of healing empyema were later diagnosed with SLE [7]. Diagnosing early pleural effusion is challenging because of its nonspecific clinical features. In this case, the patient’s presentation with unilateral pleural effusion and advanced age made malignancy and tuberculosis the top differential diagnoses, reducing our suspicion of SLE. Although bilateral pleural effusion and pleuritis in older men with SLE have been reported [8], no reports have described unilateral pleural effusion in older men.

This case underscores the difficulty of diagnosing SLE in patients of advanced age and the importance of considering a broad range of differential diagnoses, even in cases that may suggest a more common disease. However, it is important to note that the main limitation of this study is that it is a single case report, which may not fully capture the variability and complexity of SLE presentations in patients of advanced age. Further studies are necessary to validate these findings and provide more comprehensive insights.

## Conclusions

This case highlights the challenges of diagnosing SLE in older patients, particularly when such patients present with atypical symptoms, such as unilateral pleural effusion. Despite our patient’s age and initial presentation, the development of additional symptoms and positive autoantibody testing ultimately led to the correct diagnosis of SLE. This case emphasizes the importance of considering a broad differential diagnosis, including SLE, even in elderly patients presenting with conditions that may appear more common, such as pleural effusion. It also underscores the potential for unilateral pleural effusion to be an initial manifestation of SLE, even in older men.

## References

[1] Font J, Pallarés L, Cervera R, López-Soto A, Navarro M, Bosch X, Ingelmo M (1991). Systemic lupus erythematosus in the elderly: clinical and immunological characteristics. Ann Rheum Dis.

[2] Baker S, Rovira J, Campion E, Mills J (1979). Late onset systemic lupus erythematosus. Am J Med.

[3] Choi BY, Yoon MJ, Shin K, Lee YJ, Song YW (2015). Characteristics of pleural effusions in systemic lupus erythematosus: differential diagnosis of lupus pleuritis. Lupus.

[4] Gulhane S, Gangane N (2012). Detection of lupus erythematosus cells in pleural effusion: an unusual presentation of systemic lupus erythematosus. J Cytol.

[5] Yao X, Abd Hamid M, Sundaralingam A (2020). Clinical perspective and practices on pleural effusions in chronic systemic inflammatory diseases. Breathe (Sheff).

[6] Zablockis R, Petruskeviciene R, Nargela RV (2010). Causes and risk factors of pleural empyema and complicated parapneumonic pleural effusion [Article in Lithuanian]. Medicina (Kaunas).

[7] Berrou M, Fussell J, Almalouf P (2018). Lupus pleuritis can present as an exudative loculated pleural effusion mimicking empyema. Am J Respir Crit Care Med.

[8] Kita K, Asano R (2019). Late-onset systemic lupus erythematosus in a 77-year-old man with acute pleuritis. Toyama Med J.

